# Headache in General Practice: Frequency, Management, and Results of Encounter

**DOI:** 10.1155/2014/169428

**Published:** 2014-10-28

**Authors:** Thomas Frese, Henriette Druckrey, Hagen Sandholzer

**Affiliations:** Department of Primary Care, Leipzig Medical School, Philipp-Rosenthal-Straße 55, 04103 Leipzig, Germany

## Abstract

*Objective*. Headache is a common reason for consulting the general practitioner. The goal of the investigation was to characterize the headache consultation rates, the associated symptoms, the frequency of diagnostic and therapeutic procedures, and the results of the encounter of patients with headache. *Methods*. Cross-sectional data were collected from randomly selected patients during the German SESAM 2 study and compared with unpublished but publicly available data from the Dutch Transition Project. *Results*. Headache accounts for up to five percent of all general practice consultations. Women consult the general practitioner for headache twice as often as men. Physical examination and drug prescription are the most frequent procedures. Most of the patients suffer from primary headache; secondary headache is due to upper respiratory tract infections or problems of the spinal column. Dangerous courses occur in very rare cases. *Conclusion*. This work confirms the findings of earlier studies regarding the management of patients that consult the general practitioner for headache. It broadens the preexisting database since cross-sectional data regarding headache in general practice was rarely published.

## 1. Introduction

Headache is a common complaint in the general population [1]. It is under the top ten of disabling conditions on the ranking of causes of disability of the World Health Organization for both men and women and under the top five for women [2]. Most people never consult a doctor for headache [3]. It accounts for around 1.5 percent of all visits in primary care practices [4]. Headache was found at rank 14 of the most frequent reasons for consulting general practitioners in the United States [5]. It is the general practitioner who sees the patient with headache first. Some studies suggest that management of headache patients may be improved regarding diagnosis and treatment [6–9]. Despite an extensive number of publications regarding headache, data on headache as reason for encounter in a general practice setting were mainly derived from the Study of Headache in North American Primary Care during the 1980s [4] and the Australian BEACH (Bettering the Evaluation and Care of Health) program, a continuous national study of general practice that started in 1998 [10–12].

The present investigation was performed to characterize the consultation rates, the management, the differential diagnoses, and the significantly frequent comorbidities of patients encountering a typical German general practice setting. The registration of the contacts for headache was a minor part of the whole SESAM 2 study. Furthermore the results of the SESAM 2 study should be compared with the outcome of the Dutch Transition Project [13] in order to broaden the databases for this relevant context.

## 2. Methods

*SESAM 2 Study*. The Saxon Society of General Medicine (SGAM) contacted all general practitioners in Saxony by post. They received no incentive for the participation. Of the 2,510 physicians contacted, 270 general practitioners agreed to participate and 209 cooperated during the complete duration (one year). Cross-sectional data were collected from October 1, 1999, to September 30, 2000. Case recording was carried out on one day a week (Monday to Friday; either morning or afternoon consultation hours), chosen at random by the study designers, and allocated to the general practitioners. Data were collected from one of ten consecutive consulting patients. Multiple recording of the same patient was avoided. House calls were not considered. A total of 8,877 patients were included.

A standardised data collection form was used. It was developed by general practitioners (Leipzig Medical School and Saxon Society of General Medicine). The form was tested and evaluated during a pilot trial (SESAM 1). Each patient's reasons for consultation, symptoms, diagnostic procedures, recent diagnoses, and general morbidity were documented as well as therapeutic procedures. As far as possible, data was documented verbatim (according to the study instructions): either as told by the patients (e.g., reasons for consultation) or in the words of the physician (e.g., chronic diagnoses). Due to the random selection of patients, the information was documented in a reasonably short time. Only completely filled-in forms were considered.

As described elsewhere, the SESAM 2 study provides independent cross-sectional data from a typical primary care setting [14, 15]. Because total morbidity was estimated there is no selection bias or attention bias towards a single condition and the data can be assumed to be representative. The 1987 version of the International Classification of Primary Care (ICPC) was used to encode the reason for the encounter [16]. The SESAM 2 data was compared to those of two other investigations. Unpublished, but publicly available, data from the Transition Project (described by Lamberts and Okkes [13, 17]) was analysed (total estimation of patients of about 20 Dutch general practitioners; 1985 to 2003). Software containing the data and an integrated analysing tool are available at http://www.transitieproject.nl/.

*Statistical Analyses*. Data was analysed using Statistical Packages for Social Sciences (SPSS 15.0; SPSS Inc., Chicago, USA). As indicated, data was compared using Fisher's exact test. Differences were stated as statistically significant for *P* < 0.05.

## 3. Results

In the SESAM 2 study 8,877 patients (of whom 5,050 (56.9 percent) were female) were reported from 209 general practitioners, and 13,632 reasons for consultation were encoded. Two hundred and thirty-one patients (2.6 percent) consulted the general practitioner because of headache; 79 (34.2 percent) of those were male. The male to female ratio was 1 : 1.9. The consultation rate was highest for patients aged 15 to 44 years and decreased with lowest numbers (0.5 percent) in old age (>75 years). The mean age was 41.5 years. The age distribution of the patients is shown in Table 1. Symptoms that were significantly associated with headache are presented in Table 2. Almost 90 percent of the patients looking for medical advice for headache underwent a physical examination (Table 3). Other diagnostic procedures contained referrals for electroencephalograms, magnet resonance imaging, X-rays (paranasal sinuses), ultrasound, and cranial computed tomography scans. The physicians' actions towards their headache patients were mainly prescribing medication and performing follow-up consultations (Table 4). In 0.9 percent of cases, patients were hospitalised. Other therapies included acupuncture, bed rest, chirotherapy, diet, dietary advice, injections, household remedies, wet packs, manual therapy, neural therapy, cardiac training group, and referral to dentist. The results of encounter with a statistically significantly association to headache are given in Table 5. Diagnoses of the respiratory system were given in 32.5 percent of cases; 22.1 percent of the headaches were attributed to diseases of the nervous system. The most frequent diagnoses for patients with headache (Table 6). The majority of the possible dangerous courses were related to trauma or injury of the head or cervical spine (4.7 percent of the headache patients) or to acute complicated empyema of the paranasal sinuses (1.7 percent). Suspected other diseases of the circulatory system (0.9 percent), cerebrovascular diseases (0.4 percent), neoplasms of the central nervous system (0.4 percent), and calenture (0.4 percent) were rare.

For the Dutch Transition Project a total of 149,238 patients were listed as active and 219,596 consultations were assessed. 8,050 patients (5.4 percent of all patients) consulted for headache. Mostly patients between the age of 15 and 64 were looking for medical advice for headache (Table 1). The male to female ratio was 1 : 1.9. There were 9,865 consultations for headache. The top five accompanying symptoms were neck complaints (*n* = 508), weakness (*n* = 369), vertigo (*n* = 309), fever (*n* = 263), and cough (*n* = 291). Within the Dutch Transition Project 19,076 diagnostic or therapeutic procedures were documented in patients that consulted because of headache. As summarized in Table 3, physical examinations were the most common diagnostic procedures. The examinations were performed regarding the nervous system (*n* = 2,841), the respiratory tract (*n* = 1,781), the musculoskeletal system (*n* = 1,177), the cardiovascular system (*n* = 765), or unspecified/general symptoms (*n* = 738). Examination towards psychiatric problems and ear or eye problems was less frequently performed. As presented in Table 4, the most common therapeutic procedures were the prescription of drugs and giving advice. The distribution of therapeutic procedures among different ICPC chapters was similar to those of the diagnostic procedures. The most frequently chosen results of encounter are presented in Table 5. Underlying dangerous courses were rare in the Dutch Transition Project.

## 4. Discussion

Our findings demonstrated that headache frequently appeared as primary headache. In a general practice setting, secondary headache was linked to respiratory tract infections and problems of the cervical spine. Dangerous courses were rare.

The frequency of patients consulting their general practitioner because of headache varies from 1.5 percent (Study of Headache in North American Primary Care), 1.9 percent (Australian BEACH study), and 2.6 percent (German SESAM 2 study) to 5.4 percent in the Dutch Transition Project [4, 12]. These rates are comparable and unlikely influenced by an attention bias regarding headache since the SESAM 2 study, the BEACH study, and the Transition Project did not solely focus on headache as reason for encounter. According to our findings and the work of others [4, 18, 19], headache seems to be most common in the middle aged population between 15 and 64 years of age. It is more common among females. The male to female ratio was reported to be 1 : 1.1 [19] to 1 : 2.6 [20]. We found a ratio of 1 : 1.9 for both the German SESAM 2 study and the Dutch Transition Project.

The documentation of data by the respective general practitioners is one weakness of the German SESAM 2 study as well as of the Dutch Transition Project. The inclusion of the patients into the investigations was not blinded and the data may be influenced by socially desirable behaviour. However, as stated above, in the German SESAM 2 study and the Dutch Transition Project all reasons of encounter were considered, not only headache. Thereby the procedures are comparable between different reasons of encounter within these two investigations. For most of the diagnostic or therapeutic procedures similar frequencies were documented (Table 4) and additionally these frequencies confirm reported data from the Study of Headache in North American Primary Care, the Australian BEACH study, and others [4, 12, 21]. The reported referral rates (primary care and secondary care) were remarkably high in the SESAM 2 study and may be influenced by socially desirable behaviour and resulting intensified diagnostic efforts. As in the Dutch Transition Project other studies showed referral rates to specialist care of 2 to 3 percent [20].

Our findings strongly suggest that, in a daily general practice setting, headache is an accompanying symptom of infectious diseases and complaints of the spinal column (Table 5). This was also reported by others [4, 19, 22, 23]. A combination of headache with hypertension and neurological problems was described in other general practice studies [19, 22], although this could not completely be confirmed by our results. However, headache may also be a symptom caused by a systemic disease or a side effect of drug therapy [19, 24]. Although the Transition Project documented that the cause of the complaints of 0.9 percent of patients with headache was side effects of medication in proper dose, sufficient information about the medication of the included patients was not provided. Comparable to other studies, only a few dangerous courses were recorded [9, 19, 22, 23].

## 5. Conclusion

Headache is a common reason for consulting the general practitioner. Most headaches are primary or cannot be further specified. Dangerous courses are rare. A referral of patients is necessary if red flag signs appear or in the case of recurring headache.

## Figures and Tables

**Table 1:** The distribution of headache patients (pd) on different age groups and consultation prevalence (cp) of headache in these age groups in the German SESAM 2 study (total *n* = 8,877) and the Dutch Transition Project (total *n* = 149,238).

Age [years]	SESAM 2 (*n* = 231)		DTP (*n* = 8,050)	
	pd [%]	cp [%]	pd [%]	cp [%]
<4	0.4	0.9	0.9	0.7
5 to 14	0.9	0.7	9.5	5.3
15 to 24	21.1	5.5	14.6	7.2
25 to 44	35.1	4.4	38.8	6.9
45 to 64	32.0	2.6	22.7	5.6
65 to 74	7.8	1.1	8.0	4.0
>75	2.6	0.5	5.5	3.0

pd: patient distribution, refers to the group of headache patients.

cp: consultation prevalence, refers to all patients of the same age group within the study population.

**Table 2:** Comparison of the number [*n*] and percentage [%] of accompanying symptoms^*^ in general practice patients with and without headache (SESAM 2 study).

Accompanying symptoms	Headache		Without headache		*P* (Fisher)
	*n* = 231		*n* = 8, 646		
	*n*	%	*n*	%	
Fever	24	10.4	349	4.0	0.000
Coryza	23	10.0	195	2.3	0.000
Cough	22	9.5	92	1.1	0.036
Throat complaint	13	5.6	286	3.3	0.063
Weakness/tiredness	12	5.2	172	2.0	0.003
Nausea	11	4.8	74	0.9	0.000
Neck pain	9	3.9	157	1.8	0.041
Vertigo	8	3.5	44	0.5	0.000
Dysphagia	3	1.3	20	0.2	0.021
Tinnitus	3	1.3	13	0.2	0.008
Unspecified symptoms of the nervous system	3	1.3	9	0.1	0.003

^*^Further accompanying symptoms of patients with headache omitted from the table because of lacking significance: visual floaters/spots, abnormal eye sensation, abnormal eye appearance, vomiting, diarrhea, back pain, shoulder pain, reduced activity, colic, stomach ache, constipation, pyrosis, teeth problems, eye pain, otalgia, pulse irregularities, other chest pain, lumbago without emanation, knee pain, muscle pain fibrositis, unspecific pain in several joints, unspecific pain of the musculoskeletal system, paresthesia in fingers, feet, and toes, disturbances of smell and taste, acute stress reaction, feeling/behaving irritable/angry, shortness of breath, nosebleed, sinus complaint, other symptoms of the respiratory tract, acute laryngitis, tracheitis, localised rash, laceration/cut, other skin symptoms, dysuria, and hematuria.

**Table 3:** Actions taken by the physician [%] in the German SESAM 2 study (*n* = 231 patients) and the Dutch Transition Project (*n* = 8,050 patients, *n* = 9,865 consultations) to diagnose headache. Procedures that are not explicitly diagnostic were also registered in Table 4.

Physician's action	SESAM 2	DTP
Physical examination	90.5	85.1
Follow-up consultation	69.3	N/A
Laboratory investigations	13.4	6.9
Referral	15.2	1.2 (pc^*^), 3.9 (sc^**^)
Other procedures	6.5	0.6
Diagnostic imaging	4.8	3.3
ECG	4.8	N/A
Hospitalization	0.9	0.3

^*^pc: primary care; ^**^sc: specialized care.

**Table 4:** Actions taken by the physician [%] in the German SESAM 2 study (*n* = 231 patients) and the Dutch Transition Project (*n* = 8,050 patients, *n* = 9,865 consultations) to treat headache. Procedures that are not explicitly therapeutic were also registered in Table 3.

Physician's action	SESAM 2	DTP
Drug prescription	77.5	44.2
Follow-up consultation	69.3	N/A
Other therapy	13.0	3.1
Physicians advice	13.4	40.0
Referral	15.2	1.2 (pc^*^), 3.9 (sc^**^)
Physical therapy	15.6	8.2
Incapacity to work	45.0	N/A
Hospitalization	0.9	0.3

^*^pc: primary care; ^**^sc: specialized care.

**Table 5:** Comparison of the frequency (number [*n*] and percentage [%]) of results of encounter^*^ (“diagnosis”) in general practice patients with headache and without headache (SESAM 2 study).

Result of encounter	Headache		Without headache		*P* (Fisher)
	*n*	%	*n*	%	
Other upper respiratory infections	54	23.4	673	7.8	<0.001
Migraine/headache	46	19.9	9	0.1	<0.001
Other disease spinal column/back	35	15.2	713	8.2	0.001
Other symptoms/pathologic laboratory results^**^	18	7.8	237	2.7	<0.001
Other disease circulatory system	8	3.5	110	1.3	0.012
Fever of unknown origin	4	1.7	20	0.2	0.003
Chronic sinusitis	3	1.3	2	0.0	<0.001

^*^Further results of encounter of patients with headache omitted from the table because of lacking significance: Other bacterial diseases, herpes infection, other virus infections, other malign neoplasms of the skin, other neoplasms benign, in-situ or of unknown behaviour, iron deficiency anaemia, mental disorders by alcohol, schizophrenia and delusional disorders, affective disorders, neurotic and somatoform disorders, epilepsy, diseases of the nerves, nerve routes, plexus, other diseases of the nervous system, conjunctivitis, glaucoma, other diseases of the eyes, other diseases of the ears, essential hypertension, other hypertension, other cerebrovascular diseases, varicose of the lower extremities, acute pharyngitis and acute tonsillitis, acute laryngitis and tracheitis, flue, acute bronchitis and bronchiolitis, bronchial asthma, other diseases of the teeth and jaw, gastritis and duodenitis, other diseases of the esophagus, stomach or duodenum, other diseases of the bowels and peritoneum, other diseases of the liver, cholelithiasis and cholecystitis, chronic polyarthritis and other inflammatory polyarthropathies, other diseases of the joints, diseases of the smooth tissue, other diseases of the muscle and skeletal system and connective tissue, fractures of other parts of the extremities, luxation, spraining and distraction of specified and several body parts, other bruises specified and not otherwise specified on several body regions, other and not otherwise specified injuries with external causes, early complications of a trauma or surgical and medical treatment not otherwise specified.

^**^not otherwise specified.

**Table 6:** Frequency (%) of the most common results of encounter (“diagnosis”) of patients with a headache in the German SESAM 2 study (*n* = 231) and the Dutch Transition Project (*n* = 8,050 patients; frequencies refer to *n* = 10,023 episodes of care).

Result of encounter	SESAM 2	DTP
Upper respiratory tract infection	16.9	3.8
Migraine	12.1	5.9
Sinusitis acute/chronic	9.1	10.7
Syndromes cervical spine	9.1	2.4
Headache	6.5	27.1
Tension headache	4.8	12.0
Muscle pain/fibrositis	3.1	1.3
Concussion/other head injuries	3.0	1.4
Elevated blood pressure/hypertension	3.0	1.9
Other disease spinal column	2.6	0.3
Acute bronchitis/bronchiolitis	2.2	0.5
Acute tonsillitis	2.2	0.3
Hypotension	2.2	0.1
Neurotic/somatoform disorder	2.2	3.5^*^
Neck symptom/complaint	1.7	5.7
Sinus symptom/complaint	1.7	0.8
Vertigo/dizziness	1.7	0.6
Influenza (without pneumonia)	0.9	1.0
Other disease neurological system	0.9	1.1
General weakness/tiredness	0.4	1.3
Anemia	0.4	0.4
Depressive disorder	0.4	0.7
Other viral infections	0.4	2.4
Adverse effect of medical agent	0	1.0
Refractive disorders	0	0.5
Other muscular injury	0	0.4
Other	12.5	12.6

^*^Including acute stress reaction, work problems, feeling anxious/nervous/tense (0.7% each), other mental/psychological disorders (0.6%), hyperventilation, and hypochondriacal disorder (0.4% each).
